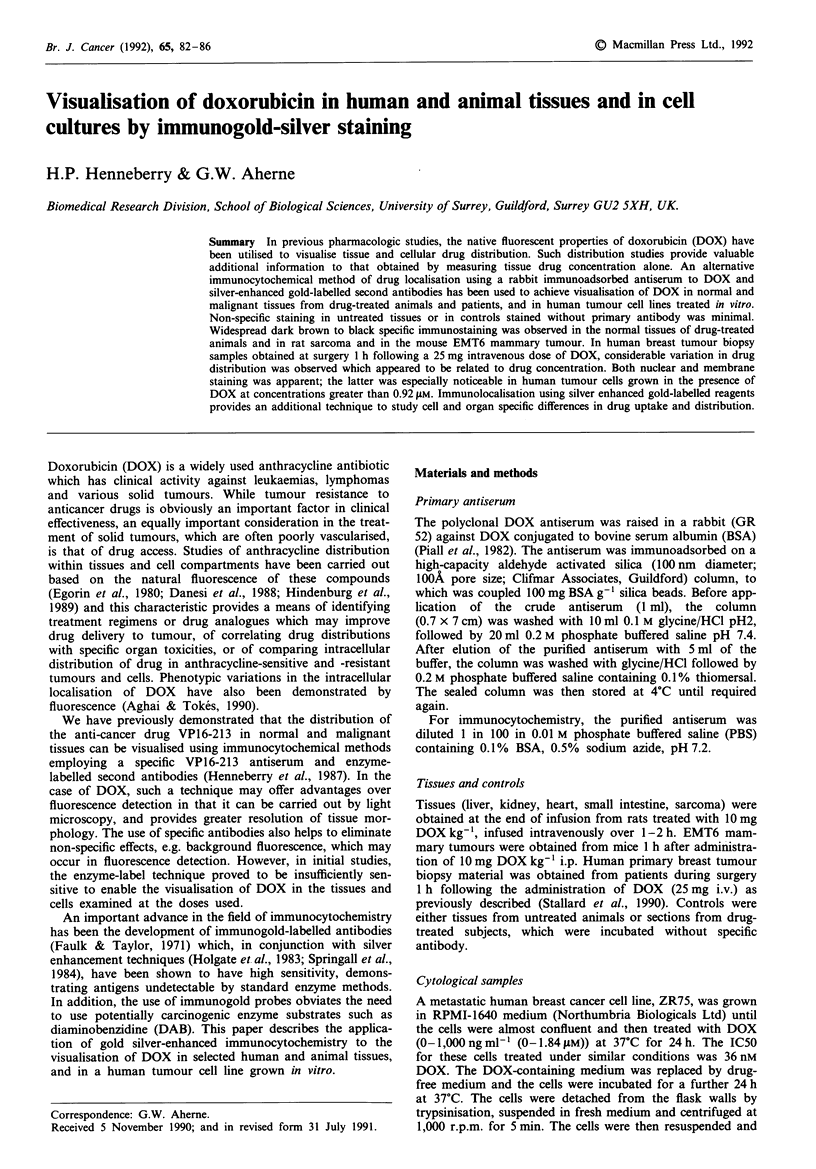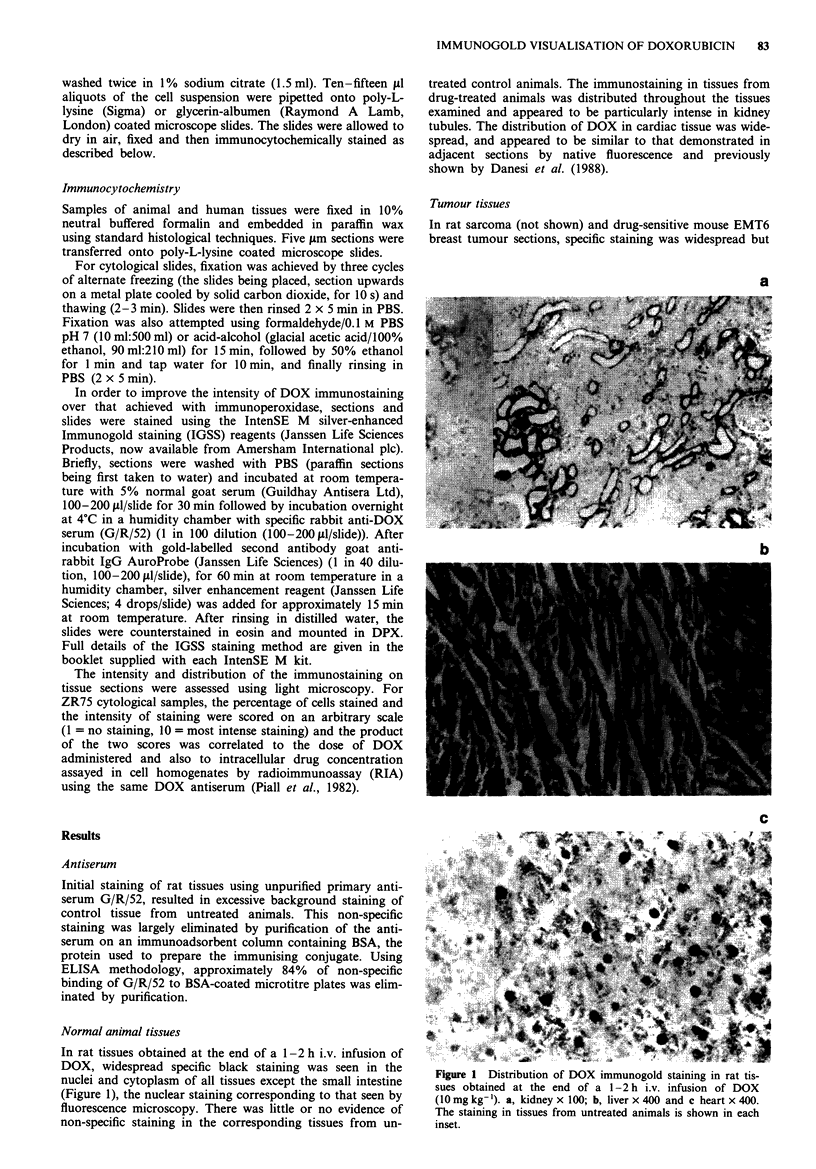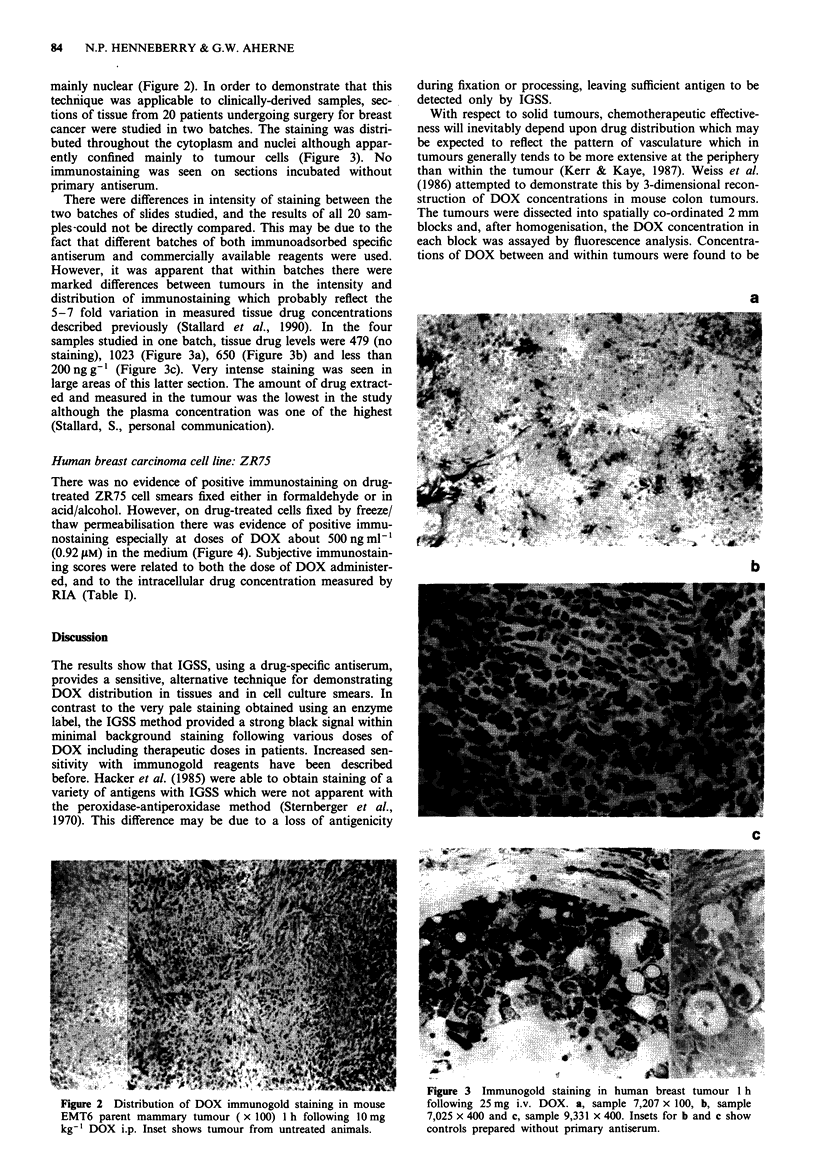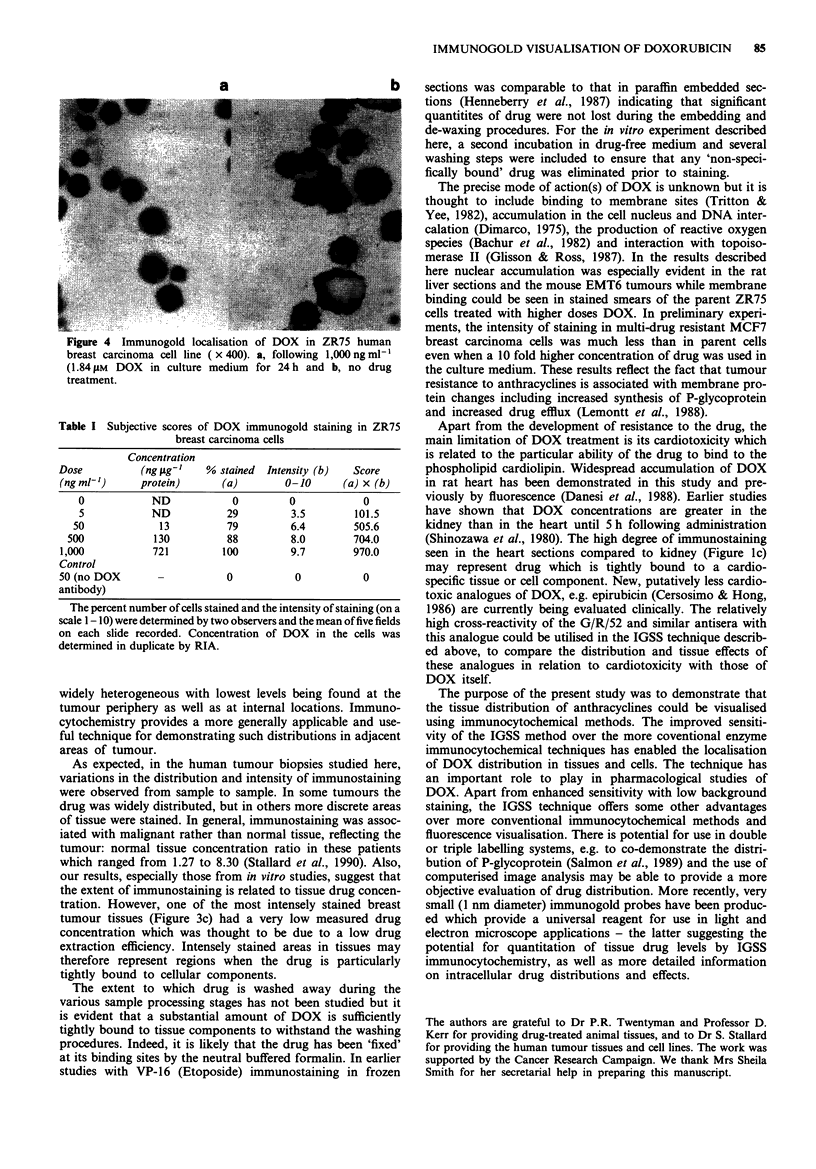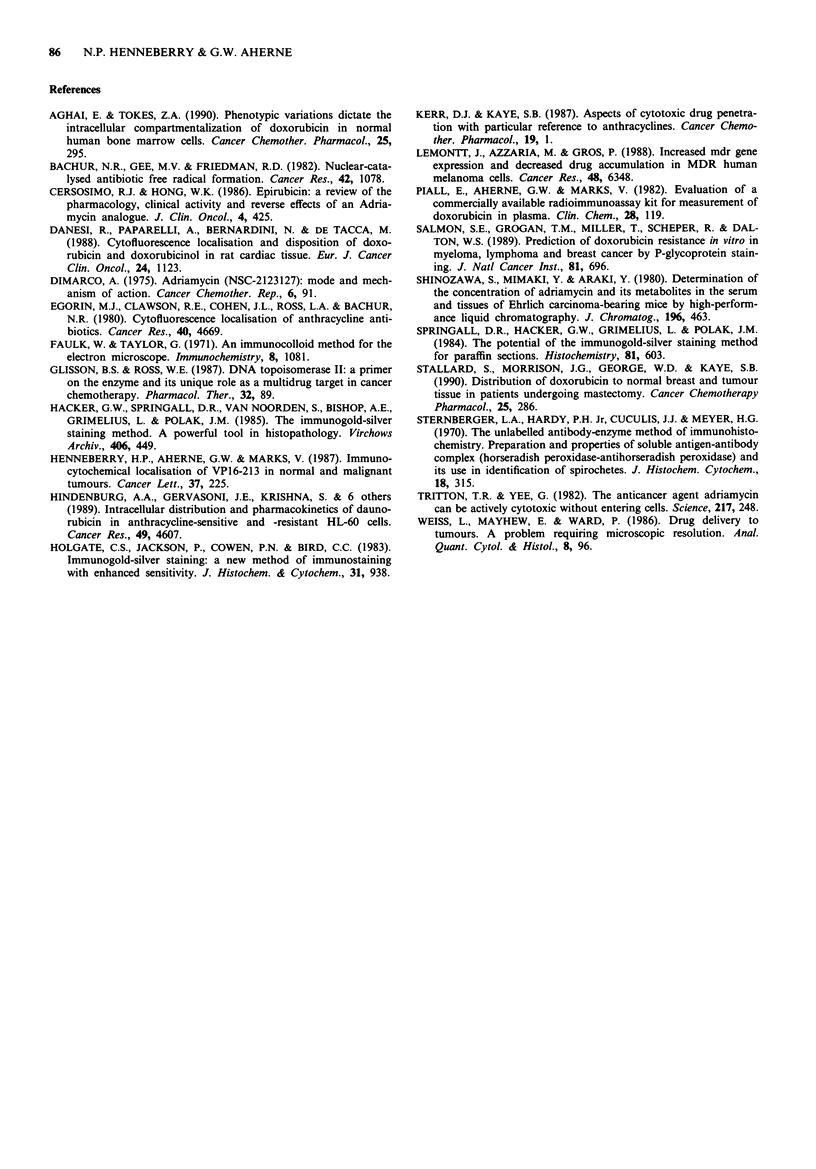# Visualisation of doxorubicin in human and animal tissues and in cell cultures by immunogold-silver staining.

**DOI:** 10.1038/bjc.1992.15

**Published:** 1992-01

**Authors:** H. P. Henneberry, G. W. Aherne

**Affiliations:** Biomedical Research Division, School of Biological Sciences, University of Surrey, Guildford, UK.

## Abstract

**Images:**


Br. J. Cancer (1992), 65, 82-86 © Macmillan Press Ltd., 1992

### Visualisation of doxorubicin in human and animal tissues and in cell cultures by immunogold-silver staining

H.P. Henneberry & G.W. Aherne

*Biomedical Research Division, School of Biological Sciences, University of Surrey, Guildford, Surrey GU2 5XH, UK.*

**Summary** In previous pharmacologic studies, the native fluorescent properties of doxorubicin (DOX) have been utilised to visualise tissue and cellular drug distribution. Such distribution studies provide valuable additional information to that obtained by measuring tissue drug concentration alone. An alternative immunocytochemical method of drug localisation using a rabbit immunoadsorbed antiserum to DOX and silver-enhanced gold-labelled second antibodies has been used to achieve visualisation of DOX in normal and malignant tissues from drug-treated animals and patients, and in human tumour cell lines treated *in vitro*. Non-specific staining in untreated tissues or in controls stained without primary antibody was minimal. Widespread dark brown to black specific immunostaining was observed in the normal tissues of drug-treated animals and in rat sarcoma and in the mouse EMT6 mammary tumour. In human breast tumour biopsy samples obtained at surgery 1 h following a 25 mg intravenous dose of DOX, considerable variation in drug distribution was observed which appeared to be related to drug concentration. Both nuclear and membrane staining was apparent; the latter was especially noticeable in human tumour cells grown in the presence of DOX at concentrations greater than 0.92 μM. Immunolocalisation using silver enhanced gold-labelled reagents provides an additional technique to study cell and organ specific differences in drug uptake and distribution.

Doxorubicin (DOX) is a widely used anthracycline antibiotic which has clinical activity against leukaemias, lymphomas and various solid tumours. While tumour resistance to anticancer drugs is obviously an important factor in clinical effectiveness, an equally important consideration in the treatment of solid tumours, which are often poorly vascularised, is that of drug access. Studies of anthracycline distribution within tissues and cell compartments have been carried out based on the natural fluorescence of these compounds (Egorin *et al.*, 1980; Danesi *et al.*, 1988; Hindenburg *et al.*, 1989) and this characteristic provides a means of identifying treatment regimens or drug analogues which may improve drug delivery to tumour, of correlating drug distributions with specific organ toxicities, or of comparing intracellular distribution of drug in anthracycline-sensitive and -resistant tumours and cells. Phenotypic variations in the intracellular localisation of DOX have also been demonstrated by fluorescence (Aghai & Tokés, 1990).

We have previously demonstrated that the distribution of the anti-cancer drug VP16-213 in normal and malignant tissues can be visualised using immunocytochemical methods employing a specific VP16-213 antiserum and enzyme-labelled second antibodies (Henneberry *et al.*, 1987). In the case of DOX, such a technique may offer advantages over fluorescence detection in that it can be carried out by light microscopy, and provides greater resolution of tissue morphology. The use of specific antibodies also helps to eliminate non-specific effects, e.g. background fluorescence, which may occur in fluorescence detection. However, in initial studies, the enzyme-label technique proved to be insufficiently sensitive to enable the visualisation of DOX in the tissues and cells examined at the doses used.

An important advance in the field of immunocytochemistry has been the development of immunogold-labelled antibodies (Faulk & Taylor, 1971) which, in conjunction with silver enhancement techniques (Holgate *et al.*, 1983; Springall *et al.*, 1984), have been shown to have high sensitivity, demonstrating antigens undetectable by standard enzyme methods. In addition, the use of immunogold probes obviates the need to use potentially carcinogenic enzyme substrates such as diaminobenzidine (DAB). This paper describes the application of gold silver-enhanced immunocytochemistry to the visualisation of DOX in selected human and animal tissues, and in a human tumour cell line grown *in vitro*.

Correspondence: G.W. Aherne.
Received 5 November 1990; and in revised form 31 July 1991.

### Materials and methods

### *Primary antiserum*

The polyclonal DOX antiserum was raised in a rabbit (GR 52) against DOX conjugated to bovine serum albumin (BSA) (Piall *et al.*, 1982). The antiserum was immunoadsorbed on a high-capacity aldehyde activated silica (100 nm diameter; 100Å pore size; Clifmar Associates, Guildford) column, to which was coupled 100 mg BSA $g^{-1}$ silica beads. Before application of the crude antiserum (1 ml), the column (0.7 × 7 cm) was washed with 10 ml 0.1 M glycine/HCl pH2, followed by 20 ml 0.2 M phosphate buffered saline pH 7.4. After elution of the purified antiserum with 5 ml of the buffer, the column was washed with glycine/HCl followed by 0.2 M phosphate buffered saline containing 0.1% thiomersal. The sealed column was then stored at 4°C until required again.

For immunocytochemistry, the purified antiserum was diluted 1 in 100 in 0.01 M phosphate buffered saline (PBS) containing 0.1% BSA, 0.5% sodium azide, pH 7.2.

### *Tissues and controls*

Tissues (liver, kidney, heart, small intestine, sarcoma) were obtained at the end of infusion from rats treated with 10 mg DOX $kg^{-1}$, infused intravenously over 1-2 h. EMT6 mammary tumours were obtained from mice 1 h after administration of 10 mg DOX $kg^{-1}$ i.p. Human primary breast tumour biopsy material was obtained from patients during surgery 1 h following the administration of DOX (25 mg i.v.) as previously described (Stallard *et al.*, 1990). Controls were either tissues from untreated animals or sections from drug-treated subjects, which were incubated without specific antibody.

### *Cytological samples*

A metastatic human breast cancer cell line, ZR75, was grown in RPMI-1640 medium (Northumbria Biologicals Ltd) until the cells were almost confluent and then treated with DOX (0-1,000 ng $ml^{-1}$ (0-1.84 μM)) at 37°C for 24 h. The IC50 for these cells treated under similar conditions was 36 nM DOX. The DOX-containing medium was replaced by drug-free medium and the cells were incubated for a further 24 h at 37°C. The cells were detached from the flask walls by trypsinisation, suspended in fresh medium and centrifuged at 1,000 r.p.m. for 5 min. The cells were then resuspended and washed twice in 1% sodium citrate (1.5 ml). Ten–fifteen µl aliquots of the cell suspension were pipetted onto poly-L-lysine (Sigma) or glycerin-albumen (Raymond A Lamb, London) coated microscope slides. The slides were allowed to dry in air, fixed and then immunocytochemically stained as described below.

*Immunocytochemistry*

Samples of animal and human tissues were fixed in 10% neutral buffered formalin and embedded in paraffin wax using standard histological techniques. Five µm sections were transferred onto poly-L-lysine coated microscope slides.

For cytological slides, fixation was achieved by three cycles of alternate freezing (the slides being placed, section upwards on a metal plate cooled by solid carbon dioxide, for 10 s) and thawing (2–3 min). Slides were then rinsed 2 × 5 min in PBS. Fixation was also attempted using formaldehyde/0.1 M PBS pH 7 (10 ml:500 ml) or acid-alcohol (glacial acetic acid/100% ethanol, 90 ml:210 ml) for 15 min, followed by 50% ethanol for 1 min and tap water for 10 min, and finally rinsing in PBS (2 × 5 min).

In order to improve the intensity of DOX immunostaining over that achieved with immunoperoxidase, sections and slides were stained using the IntenSE M silver-enhanced Immunogold staining (IGSS) reagents (Janssen Life Sciences Products, now available from Amersham International plc). Briefly, sections were washed with PBS (paraffin sections being first taken to water) and incubated at room temperature with 5% normal goat serum (Guildhay Antisera Ltd), 100–200 µl/slide for 30 min followed by incubation overnight at 4°C in a humidity chamber with specific rabbit anti-DOX serum (G/R/52) (1 in 100 dilution (100–200 µl/slide)). After incubation with gold-labelled second antibody goat anti-rabbit IgG AuroProbe (Janssen Life Sciences) (1 in 40 dilution, 100–200 µl/slide), for 60 min at room temperature in a humidity chamber, silver enhancement reagent (Janssen Life Sciences; 4 drops/slide) was added for approximately 15 min at room temperature. After rinsing in distilled water, the slides were counterstained in eosin and mounted in DPX. Full details of the IGSS staining method are given in the booklet supplied with each IntenSE M kit.

The intensity and distribution of the immunostaining on tissue sections were assessed using light microscopy. For ZR75 cytological samples, the percentage of cells stained and the intensity of staining were scored on an arbitrary scale (1 = no staining, 10 = most intense staining) and the product of the two scores was correlated to the dose of DOX administered and also to intracellular drug concentration assayed in cell homogenates by radioimmunoassay (RIA) using the same DOX antiserum (Piall *et al.*, 1982).

### Results

*Antiserum*

Initial staining of rat tissues using unpurified primary antiserum G/R/52, resulted in excessive background staining of control tissue from untreated animals. This non-specific staining was largely eliminated by purification of the antiserum on an immunoadsorbent column containing BSA, the protein used to prepare the immunising conjugate. Using ELISA methodology, approximately 84% of non-specific binding of G/R/52 to BSA-coated microtitre plates was eliminated by purification.

*Normal animal tissues*

In rat tissues obtained at the end of a 1–2 h i.v. infusion of DOX, widespread specific black staining was seen in the nuclei and cytoplasm of all tissues except the small intestine (Figure 1), the nuclear staining corresponding to that seen by fluorescence microscopy. There was little or no evidence of non-specific staining in the corresponding tissues from untreated control animals. The immunostaining in tissues from drug-treated animals was distributed throughout the tissues examined and appeared to be particularly intense in kidney tubules. The distribution of DOX in cardiac tissue was widespread, and appeared to be similar to that demonstrated in adjacent sections by native fluorescence and previously shown by Danesi *et al.* (1988).

*Tumour tissues*

In rat sarcoma (not shown) and drug-sensitive mouse EMT6 breast tumour sections, specific staining was widespread but

**Figure 1** Distribution of DOX immunogold staining in rat tissues obtained at the end of a 1–2 h i.v. infusion of DOX (10 mg $kg^{-1}$). **a**, kidney × 100; **b**, liver × 400 and **c** heart × 400. The staining in tissues from untreated animals is shown in each inset.

mainly nuclear (Figure 2). In order to demonstrate that this technique was applicable to clinically-derived samples, sections of tissue from 20 patients undergoing surgery for breast cancer were studied in two batches. The staining was distributed throughout the cytoplasm and nuclei although apparently confined mainly to tumour cells (Figure 3). No immunostaining was seen on sections incubated without primary antiserum.

There were differences in intensity of staining between the two batches of slides studied, and the results of all 20 samples could not be directly compared. This may be due to the fact that different batches of both immunoadsorbed specific antiserum and commercially available reagents were used. However, it was apparent that within batches there were marked differences between tumours in the intensity and distribution of immunostaining which probably reflect the 5–7 fold variation in measured tissue drug concentrations described previously (Stallard *et al.*, 1990). In the four samples studied in one batch, tissue drug levels were 479 (no staining), 1023 (Figure 3a), 650 (Figure 3b) and less than 200 ng g$^{-1}$ (Figure 3c). Very intense staining was seen in large areas of this latter section. The amount of drug extracted and measured in the tumour was the lowest in the study although the plasma concentration was one of the highest (Stallard, S., personal communication).

*Human breast carcinoma cell line: ZR75*

There was no evidence of positive immunostaining on drug-treated ZR75 cell smears fixed either in formaldehyde or in acid/alcohol. However, on drug-treated cells fixed by freeze/thaw permeabilisation there was evidence of positive immunostaining especially at doses of DOX about 500 ng ml$^{-1}$ (0.92 μM) in the medium (Figure 4). Subjective immunostaining scores were related to both the dose of DOX administered, and to the intracellular drug concentration measured by RIA (Table I).

### Discussion

The results show that IGSS, using a drug-specific antiserum, provides a sensitive, alternative technique for demonstrating DOX distribution in tissues and in cell culture smears. In contrast to the very pale staining obtained using an enzyme label, the IGSS method provided a strong black signal within minimal background staining following various doses of DOX including therapeutic doses in patients. Increased sensitivity with immunogold reagents have been described before. Hacker *et al.* (1985) were able to obtain staining of a variety of antigens with IGSS which were not apparent with the peroxidase-antiperoxidase method (Sternberger *et al.*, 1970). This difference may be due to a loss of antigenicity during fixation or processing, leaving sufficient antigen to be detected only by IGSS.

With respect to solid tumours, chemotherapeutic effectiveness will inevitably depend upon drug distribution which may be expected to reflect the pattern of vasculature which in tumours generally tends to be more extensive at the periphery than within the tumour (Kerr & Kaye, 1987). Weiss *et al.* (1986) attempted to demonstrate this by 3-dimensional reconstruction of DOX concentrations in mouse colon tumours. The tumours were dissected into spatially co-ordinated 2 mm blocks and, after homogenisation, the DOX concentration in each block was assayed by fluorescence analysis. Concentrations of DOX between and within tumours were found to be

**Figure 2** Distribution of DOX immunogold staining in mouse EMT6 parent mammary tumour (× 100) 1 h following 10 mg kg$^{-1}$ DOX i.p. Inset shows tumour from untreated animals.

**Figure 3** Immunogold staining in human breast tumour 1 h following 25 mg i.v. DOX. **a**, sample 7,207 × 100, **b**, sample 7,025 × 400 and **c**, sample 9,331 × 400. Insets for **b** and **c** show controls prepared without primary antiserum.

**Figure 4** Immunogold localisation of DOX in ZR75 human breast carcinoma cell line ( × 400). **a**, following 1,000 ng ml$^{-1}$ (1.84 μM DOX in culture medium for 24 h and **b**, no drug treatment.

**Table I** Subjective scores of DOX immunogold staining in ZR75 breast carcinoma cells

| *Dose (ng ml$^{-1}$)* | *Concentration (ng μg$^{-1}$ protein)* | *% stained (a)* | *Intensity (b) 0–10* | *Score (a) × (b)* |
|---|---|---|---|---|
| 0 | ND | 0 | 0 | 0 |
| 5 | ND | 29 | 3.5 | 101.5 |
| 50 | 13 | 79 | 6.4 | 505.6 |
| 500 | 130 | 88 | 8.0 | 704.0 |
| 1,000 | 721 | 100 | 9.7 | 970.0 |
| *Control* | | | | |
| 50 (no DOX antibody) | – | 0 | 0 | 0 |

The percent number of cells stained and the intensity of staining (on a scale 1 – 10) were determined by two observers and the mean of five fields on each slide recorded. Concentration of DOX in the cells was determined in duplicate by RIA.

widely heterogeneous with lowest levels being found at the tumour periphery as well as at internal locations. Immunocytochemistry provides a more generally applicable and useful technique for demonstrating such distributions in adjacent areas of tumour.

As expected, in the human tumour biopsies studied here, variations in the distribution and intensity of immunostaining were observed from sample to sample. In some tumours the drug was widely distributed, but in others more discrete areas of tissue were stained. In general, immunostaining was associated with malignant rather than normal tissue, reflecting the tumour: normal tissue concentration ratio in these patients which ranged from 1.27 to 8.30 (Stallard *et al.*, 1990). Also, our results, especially those from *in vitro* studies, suggest that the extent of immunostaining is related to tissue drug concentration. However, one of the most intensely stained breast tumour tissues (Figure 3c) had a very low measured drug concentration which was thought to be due to a low drug extraction efficiency. Intensely stained areas in tissues may therefore represent regions when the drug is particularly tightly bound to cellular components.

The extent to which drug is washed away during the various sample processing stages has not been studied but it is evident that a substantial amount of DOX is sufficiently tightly bound to tissue components to withstand the washing procedures. Indeed, it is likely that the drug has been 'fixed' at its binding sites by the neutral buffered formalin. In earlier studies with VP-16 (Etoposide) immunostaining in frozen sections was comparable to that in paraffin embedded sections (Henneberry *et al.*, 1987) indicating that significant quantitites of drug were not lost during the embedding and de-waxing procedures. For the *in vitro* experiment described here, a second incubation in drug-free medium and several washing steps were included to ensure that any 'non-specifically bound' drug was eliminated prior to staining.

The precise mode of action(s) of DOX is unknown but it is thought to include binding to membrane sites (Tritton & Yee, 1982), accumulation in the cell nucleus and DNA intercalation (Dimarco, 1975), the production of reactive oxygen species (Bachur *et al.*, 1982) and interaction with topoisomerase II (Glisson & Ross, 1987). In the results described here nuclear accumulation was especially evident in the rat liver sections and the mouse EMT6 tumours while membrane binding could be seen in stained smears of the parent ZR75 cells treated with higher doses DOX. In preliminary experiments, the intensity of staining in multi-drug resistant MCF7 breast carcinoma cells was much less than in parent cells even when a 10 fold higher concentration of drug was used in the culture medium. These results reflect the fact that tumour resistance to anthracyclines is associated with membrane protein changes including increased synthesis of P-glycoprotein and increased drug efflux (Lemontt *et al.*, 1988).

Apart from the development of resistance to the drug, the main limitation of DOX treatment is its cardiotoxicity which is related to the particular ability of the drug to bind to the phospholipid cardiolipin. Widespread accumulation of DOX in rat heart has been demonstrated in this study and previously by fluorescence (Danesi *et al.*, 1988). Earlier studies have shown that DOX concentrations are greater in the kidney than in the heart until 5 h following administration (Shinozawa *et al.*, 1980). The high degree of immunostaining seen in the heart sections compared to kidney (Figure 1c) may represent drug which is tightly bound to a cardio-specific tissue or cell component. New, putatively less cardiotoxic analogues of DOX, e.g. epirubicin (Cersosimo & Hong, 1986) are currently being evaluated clinically. The relatively high cross-reactivity of the G/R/52 and similar antisera with this analogue could be utilised in the IGSS technique described above, to compare the distribution and tissue effects of these analogues in relation to cardiotoxicity with those of DOX itself.

The purpose of the present study was to demonstrate that the tissue distribution of anthracyclines could be visualised using immunocytochemical methods. The improved sensitivity of the IGSS method over the more coventional enzyme immunocytochemical techniques has enabled the localisation of DOX distribution in tissues and cells. The technique has an important role to play in pharmacological studies of DOX. Apart from enhanced sensitivity with low background staining, the IGSS technique offers some other advantages over more conventional immunocytochemical methods and fluorescence visualisation. There is potential for use in double or triple labelling systems, e.g. to co-demonstrate the distribution of P-glycoprotein (Salmon *et al.*, 1989) and the use of computerised image analysis may be able to provide a more objective evaluation of drug distribution. More recently, very small (1 nm diameter) immunogold probes have been produced which provide a universal reagent for use in light and electron microscope applications – the latter suggesting the potential for quantitation of tissue drug levels by IGSS immunocytochemistry, as well as more detailed information on intracellular drug distributions and effects.

The authors are grateful to Dr P.R. Twentyman and Professor D. Kerr for providing drug-treated animal tissues, and to Dr S. Stallard for providing the human tumour tissues and cell lines. The work was supported by the Cancer Research Campaign. We thank Mrs Sheila Smith for her secretarial help in preparing this manuscript.

### References

AGHAI, E. & TOKES, Z.A. (1990). Phenotypic variations dictate the intracellular compartmentalization of doxorubicin in normal human bone marrow cells. *Cancer Chemother. Pharmacol.*, **25**, 295.

BACHUR, N.R., GEE, M.V. & FRIEDMAN, R.D. (1982). Nuclear-catalysed antibiotic free radical formation. *Cancer Res.*, **42**, 1078.

CERSOSIMO, R.J. & HONG, W.K. (1986). Epirubicin: a review of the pharmacology, clinical activity and reverse effects of an Adriamycin analogue. *J. Clin. Oncol.*, **4**, 425.

DANESI, R., PAPARELLI, A., BERNARDINI, N. & DE TACCA, M. (1988). Cytofluorescence localisation and disposition of doxorubicin and doxorubicinol in rat cardiac tissue. *Eur. J. Cancer Clin. Oncol.*, **24**, 1123.

DIMARCO, A. (1975). Adriamycin (NSC-2123127): mode and mechanism of action. *Cancer Chemother. Rep.*, **6**, 91.

EGORIN, M.J., CLAWSON, R.E., COHEN, J.L., ROSS, L.A. & BACHUR, N.R. (1980). Cytofluorescence localisation of anthracycline antibiotics. *Cancer Res.*, **40**, 4669.

FAULK, W. & TAYLOR, G. (1971). An immunocolloid method for the electron microscope. *Immunochemistry*, **8**, 1081.

GLISSON, B.S. & ROSS, W.E. (1987). DNA topoisomerase II: a primer on the enzyme and its unique role as a multidrug target in cancer chemotherapy. *Pharmacol. Ther.*, **32**, 89.

HACKER, G.W., SPRINGALL, D.R., VAN NOORDEN, S., BISHOP, A.E., GRIMELIUS, L. & POLAK, J.M. (1985). The immunogold-silver staining method. A powerful tool in histopathology. *Virchows Archiv.*, **406**, 449.

HENNEBERRY, H.P., AHERNE, G.W. & MARKS, V. (1987). Immunocytochemical localisation of VP16-213 in normal and malignant tumours. *Cancer Lett.*, **37**, 225.

HINDENBURG, A.A., GERVASONI, J.E., KRISHNA, S. & 6 others (1989). Intracellular distribution and pharmacokinetics of daunorubicin in anthracycline-sensitive and -resistant HL-60 cells. *Cancer Res.*, **49**, 4607.

HOLGATE, C.S., JACKSON, P., COWEN, P.N. & BIRD, C.C. (1983). Immunogold-silver staining: a new method of immunostaining with enhanced sensitivity. *J. Histochem. & Cytochem.*, **31**, 938.

KERR, D.J. & KAYE, S.B. (1987). Aspects of cytotoxic drug penetration with particular reference to anthracyclines. *Cancer Chemother. Pharmacol.*, **19**, 1.

LEMONTT, J., AZZARIA, M. & GROS, P. (1988). Increased mdr gene expression and decreased drug accumulation in MDR human melanoma cells. *Cancer Res.*, **48**, 6348.

PIALL, E., AHERNE, G.W. & MARKS, V. (1982). Evaluation of a commercially available radioimmunoassay kit for measurement of doxorubicin in plasma. *Clin. Chem.*, **28**, 119.

SALMON, S.E., GROGAN, T.M., MILLER, T., SCHEPER, R. & DALTON, W.S. (1989). Prediction of doxorubicin resistance *in vitro* in myeloma, lymphoma and breast cancer by P-glycoprotein staining. *J. Natl Cancer Inst.*, **81**, 696.

SHINOZAWA, S., MIMAKI, Y. & ARAKI, Y. (1980). Determination of the concentration of adriamycin and its metabolites in the serum and tissues of Ehrlich carcinoma-bearing mice by high-performance liquid chromatography. *J. Chromatog.*, **196**, 463.

SPRINGALL, D.R., HACKER, G.W., GRIMELIUS, L. & POLAK, J.M. (1984). The potential of the immunogold-silver staining method for paraffin sections. *Histochemistry*, **81**, 603.

STALLARD, S., MORRISON, J.G., GEORGE, W.D. & KAYE, S.B. (1990). Distribution of doxorubicin to normal breast and tumour tissue in patients undergoing mastectomy. *Cancer Chemotherapy Pharmacol.*, **25**, 286.

STERNBERGER, L.A., HARDY, P.H. Jr, CUCULIS, J.J. & MEYER, H.G. (1970). The unlabelled antibody-enzyme method of immunohistochemistry. Preparation and properties of soluble antigen-antibody complex (horseradish peroxidase-antihorseradish peroxidase) and its use in identification of spirochetes. *J. Histochem. Cytochem.*, **18**, 315.

TRITTON, T.R. & YEE, G. (1982). The anticancer agent adriamycin can be actively cytotoxic without entering cells. *Science*, **217**, 248.

WEISS, L., MAYHEW, E. & WARD, P. (1986). Drug delivery to tumours. A problem requiring microscopic resolution. *Anal. Quant. Cytol. & Histol.*, **8**, 96.